# Plant extracellular vesicles: A novel bioactive nanoparticle for tumor therapy

**DOI:** 10.3389/fphar.2022.1006299

**Published:** 2022-09-29

**Authors:** Zhao-Lin Tan, Jing-Fei Li, Hao-Ming Luo, Yang-Yang Liu, Ye Jin

**Affiliations:** School of Pharmacy, Changchun University of Traditional Chinese Medicine, Changchun, China

**Keywords:** plant extracellular vesicles, exosomes, anti-tumor, drug delivery, isolation, non-coding RNA

## Abstract

Extracellular vesicles are tiny lipid bilayer-enclosed membrane particles, including apoptotic bodies, micro vesicles, and exosomes. Organisms of all life forms can secrete extracellular vesicles into their surrounding environment, which serve as important communication tools between cells and between cells and the environment, and participate in a variety of physiological processes. According to new evidence, plant extracellular vesicles play an important role in the regulation of transboundary molecules with interacting organisms. In addition to carrying signaling molecules (nucleic acids, proteins, metabolic wastes, etc.) to mediate cellular communication, plant cells External vesicles themselves can also function as functional molecules in the cellular microenvironment across cell boundaries. This review introduces the source and extraction of plant extracellular vesicles, and attempts to clarify its anti-tumor mechanism by summarizing the current research on plant extracellular vesicles for disease treatment. We speculate that the continued development of plant extracellular vesicle-based therapeutic and drug delivery platforms will benefit their clinical applications.

## Introduction

Cancer is a complex disease, that is, facilitated by many factors, including genetic and environmental factors. Unfortunately, the latest cancer statistics show that cancer mortality and morbidity have been increasing, and cancer remains the leading cause of death in every country worldwide (Sung et al., 2021). Chemotherapy, as the main means of clinical treatment, has achieved good results in overcoming the problem of cancer (Galmarini et al., 2012). Chemotherapy, however, does not perfectly target tumors, and often fails to completely eliminate cancer cells, and can even damage more normal cells while killing tumor cells (Behranvand et al., 2022). A new therapeutic material with stability, good target specificity and biosafety is urgently needed (Liang et al., 2021). The combined application of extracellular vesicles and chemotherapeutic agents has brought new light to cancer patients, although they were initially considered cellular debris (Pan and Johnstone, 1983; Li et al., 2022). Extracellular vesicles are bilayer-membrane lipid spheres secreted by various organisms. Exosomes are a subset of extracellular vesicles that can carry biologically active molecules such as nucleic acids, proteins and lipids (Ratajczak et al., 2006; Chen et al., 2019; Doyle and Wang, 2019; Mathieu et al., 2019). Although various types of extracellular vesicles have been identified at the first International Society for Extracellular Vesicles (ISEV) meeting, since most of the examples in this review cannot distinguish their types, this paper uses extracellular vesicles (or exosomes) to refer to them (Witwer and Thery, 2019). In mammalian cells, both normal cells and cancer cells can secrete extracellular vesicles as a tool for cell-to-cell communication (Muralidharan-Chari et al., 2010; Kuriyama et al., 2020; Mittal et al., 2020; Schweiger and Tannous, 2020; Wu et al., 2021), Information transfer through binding of vesicle membrane proteins to receptor membrane proteins and through internalization of vesicle contents by receptor cells (Menard et al., 2018; Ko and Naora, 2020; Vu et al., 2020; Tang et al., 2022). Similarly, plant multi-vesicular bodies secrete exosome-like nanoparticles (An et al., 2007), which, in addition to being involved in the formation of the plant’s own cell wall (Chukhchin et al., 2019) are also involved in plant-microbe interactions (Rutter and Innes, 2018), including plant defense and silencing fungi gene et al. (Regente et al., 2017; Cai et al., 2018; Bleackley et al., 2019; Cai et al., 2019). Transboundary regulation mediated by plant extracellular vesicles is therefore increasingly sought after by researchers. In fact, many experimental teams have successfully isolated these vesicles from plants and used them to overcome cancer as an emerging means of human disease treatment (Raimondo et al., 2015; Potesta et al., 2020; Yang et al., 2020).

## Source of plant extracellular vesicles

**FIGURE 1:**
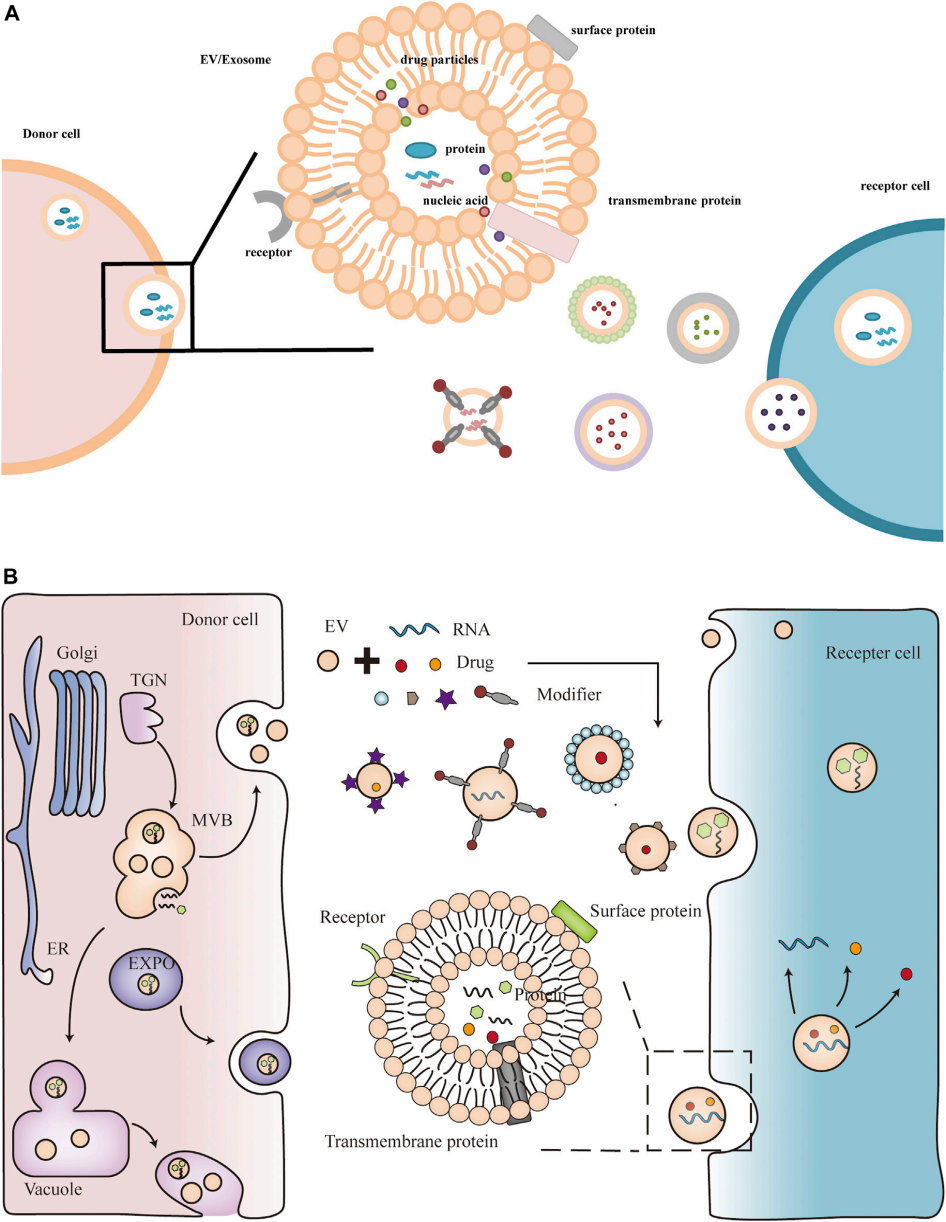
Plant extracellular vesicles contain active components such as nucleic acids and proteins. Plant extracellular vesicles can also be modified as delivery vehicles for therapeutics such as miRNAs and drugs.

In eukaryotic cells, protein secretion is usually divided into two pathways, the conventional protein secretion pathway and the unconventional protein secretion pathway (Chung and Zeng, 2017). Plant extracellular vesicles are produced mainly through unconventional protein secretion, including the alternative Golgi pathway and three pathways mediated by specific organelles: multivesicular bodies (MVB), exosome-positive organelles (EXPO), vacuolar bodies (Tse et al., 2004; Ding and Wang, 2017; Wang et al., 2018). The conventional protein secretion pathway is the transfer of proteins from the lumen of the endoplasmic reticulum (ER) to the Golgi apparatus, and then through the plasma membrane (PM), where they are released into the extracellular environment to secrete proteins. Most secreted proteins transported through the conventional protein secretion pathway have an N-terminal leader sequence, often with post-translational modifications, while the unconventional protein secretion pathway has no leader (Wang et al., 2018). In mammalian cells, EVs play an important role in mediating intercellular communication by delivering biomolecules, including proteins and RNAs, to recipient cells through the extracellular space (Menard et al., 2018). Similar to mammalian cells, there is growing evidence that plants also generate EVs that are involved in various functions. During fungal infection, ARA6 (a plant-specific Rab GTPase)-positive MVBs accumulated in plant cells and co-localized with TET8 (Tetraspanin-8) in the infected area (An et al., 2006). TET8 is structurally similar to the animal cell exosome marker CD63, therefore, TET8-labeled EVs can be regarded as plant exosomes (Kowal et al., 2016; Cai et al., 2019). This process is similar to the fusion of MVBs with the plasma membrane (PM) in animal cells to release extracellular vesicles. In addition, the SNARE (soluble N-ethylmaleimide-sensitive factor-associated protein receptor) protein SYNTAXIN121 (SYP121)/PENETRATION1 (PEN1) was also detected in plant extracellular vesicles (Meyer et al., 2009). These proteins lack typical signal peptides and may rely on unconventional protein secretion pathways to be transported out of cells, such as extracellular vesicles. EXPO is a plant-specific double-membrane structure, distinct from MVB, but also dependent on exocytosis. EXO70E2-labeled EXPO was found to fuse with PM and release internal vesicles outside the cell (Wang et al., 2010). During bacterial pathogen infection, plants initiate fusion of vacuoles with PM, releasing hydrolases and defense proteins to inhibit bacterial proliferation (Hatsugai et al., 2009). The source of plant extracellular vesicles and the way they function are shown in Figure 1.

## Extraction of plant extracellular vesicles

In addition to plant extracellular vesicles for plant growth and development, defense responses, and plant-microbe symbiosis (Regente et al., 2017; Rutter and Innes, 2018; Chukhchin et al., 2019; Ivanov et al., 2019; Zhou et al., 2021), plant extracellular vesicles have attracted attention for their potential roles in human health and disease. For example, extracellular vesicles extracted from edible fruits and vegetables have antioxidant functions, and strawberry- and blueberry-derived exosome-like nanoparticles prevent oxidative stress in human mesenchymal stromal cells and endothelial cells (De Robertis et al., 2020; Logozzi et al., 2021; Perut et al., 2021). In order to study the cargo and function of plant extracellular vesicles, isolation and purification of plant extracellular vesicles are the primary tasks. Differential centrifugation is the most commonly used method for the isolation of extracellular vesicles, which separates and removes components other than extracellular vesicles from solution in a step-by-step fashion based on differences in vesicle density and size (Carnino et al., 2019; Akbar et al., 2022). Typically, low-speed centrifugation is used to separate cellular debris, and high-speed centrifugation is used to collect extracellular vesicles by density (Thery et al., 2006; Xu et al., 2015). For example, ginseng nanovesicles were isolated from ginseng root grinding liquid by differential centrifugation and sucrose density gradient method (Cho et al., 2021). In addition, density gradient centrifugation, which can also collect extracellular vesicles based on the buoyant density of the particles, is commonly used to isolate extracellular vesicles partially isolated by centrifugation (Carnino et al., 2019). Density gradient centrifugation utilizes two methods to form gradients, a continuous density gradient or a stepwise gradient, on a sucrose pad (Konoshenko et al., 2018). For example, citrus lemon nanovesicles obtained by ultra-fast separation were purified on a 30% sucrose gradient (Raimondo et al., 2015). Corn-derived nanovesicles were obtained by ultracentrifugation of corn homogenate using a sucrose pad (Sasaki et al., 2021). In addition, based on size, precipitation and immunoaffinity capture of exosomes, ultrafiltration (UF), size exclusion chromatography (SEC), polymer precipitation, and immunoprecipitation have also been developed for the isolation of extracellular vesicles (Konoshenko et al., 2018). The separation principle of ultrafiltration is the same as that of filter membrane, and compared with ultracentrifugation, ultrafiltration takes less time (Konoshenko et al., 2018; Liangsupree et al., 2021). In particular, ultrafiltration can be alternated with successive ultracentrifugation stages (Sasaki et al., 2021). Size exclusion chromatography is a separation method based on particle size. Larger vesicles are washed out earlier because they cannot penetrate into the cavity of the column, and smaller vesicles are retained in the cavity of the column. The benefit of this approach is that it preserves the integrity of the vesicle structure (Liangsupree et al., 2021; Akbar et al., 2022). The PEG precipitation method can precipitate extracellular vesicles from the sample solution according to the solubility difference of the particles in PEG (Liangsupree et al., 2021; Akbar et al., 2022). However, due to similar solubility, some non-extracellular vesicle proteins are also precipitated. Immunoprecipitation is based on surface proteins of extracellular vesicles, such as CD63 (Carnino et al., 2019; Akbar et al., 2022). The advantage of this method is that it can specifically identify a type of extracellular vesicles, and can also better protect the integrity of the vesicles. Although the isolation methods of plant extracellular vesicles are constantly improving, the rapid, efficient and reproducible isolation of these extracellular vesicles is still challenging. Above, we briefly listed the uses and isolation methods of some plant extracellular vesicles in clinical settings, and summarized them in Tables 1, 2.

**TABLE 1:** Sources, pathways, extraction methods and applications of exosomes.

Source	Pathway	Method	Application	References
Ginseng	TLR4 MyD88	Differential centrifugation	Melanoma	Cao et al., (2019)
*Dendropanax morbifera*	MITF TYR TRP-1 TRP-2	Differential centrifugation	Melanoma	Lee et al., (2020)
Grapefruit	CCNB1 CCNB2 p21 ICAM-1	Differential centrifugation	Melanoma	Stanly et al., (2020)
*Moringa oleifera*	Bcl-2	Differential centrifugation	HeLa cell	Abdul Rahman et al., (2020)
Asparagus	Ki67 PCNA	Differential centrifugation	HCC	Zhang et al., (2021)
Lemon	Cas3 Cleaved caspase-3 TRAIL	Density gradient centrifugation	A549 SW480, SW480 LAMA84	Raimondo et al., (2015)
Corn	TNF-α	Sucrose cushion ultracentrifugation	CT26	Sasaki et al., (2021)
Garlic	p53 Bax Cas3 Cas9 Bcl-2 VEGF	Differential centrifugation	ATCC HTB-44 ATCC CCL-185	Ozkan et al., (2021)

**TABLE 2:** Exosomes as drug carriers to deliver different drugs.

Source	Pathway	Compound	Method	Application	References
Bitter melon	NLRP3 MAP30	5-FU	Electrophoresis Dialysis	OSSC	Yang et al., (2021)
Grapefruit	MHCI	miR17	Sucrose gradient centrifugation	Brain tumor	Friedmann, (2016)
Grapefruit	HER2	Doxorubicin	Ultracentrifugation	Breast cancer	Tang et al., (2020)
Grapefruit	LFA-1 CXCR1 CXCR2	Doxorubicin	Density gradient centrifugation	CT26	Wang et al., (2015)
Lemon	P-gp	Doxorubicin	Density gradient centrifugation	Ovarian cancer	Xiao et al., (2022)

## Plant extracellular vesicles as therapeutic agents against tumors

Plant extracellular vesicles contain a large amount of proteins, lipids, and miRNAs, which can act as cell messengers to transfer these biologically active substances from *in vitro* to *in vivo*, then to the lesion tissue, and finally to cells (Yim and Choi, 2016; Xiao et al., 2018). Thus, plant extracellular vesicles can mediate specific transboundary cellular or tissue responses (Zhang et al., 2012; Chin et al., 2016). Among them, plant-derived miRNAs are not only used for plant growth, development and defense responses (Xu et al., 2022), such small non-coding RNAs have also recently been used as tumor suppressor RNAs to play a role in tumor progression (Potesta et al., 2020). For example, broccoli miR159 can inhibit the growth of breast cancer cells across borders (Chin et al., 2016); olive small RNA is functionally homologous to human miR34a, and transfection of it can reduce the protein expression of hsa-miR34a mRNA targets across borders and increase different tumors Cell apoptosis and reduced proliferation (Minutolo et al., 2018); some small RNAs from maize can significantly reduce HeLa cell proliferation (Luo et al., 2017). In addition to oral absorption, the way that these plant RNAs enter the human body may also be mediated through extracellular vesicles. The molecular structure of plant extracellular vesicles is similar to that of animal extracellular vesicles, which makes plant extracellular vesicles also used as a natural therapeutic agent for disease treatment (Akers et al., 2013; Rutter and Innes, 2018). For example, exosomes isolated from wheat, asparagus, and grapefruit act as antioxidants, enhance cell viability, and promote skin cell proliferation and migration during wound healing and skin regeneration (Sahin et al., 2019; Kim et al., 2021; Savci et al., 2021). Food-derived plant exosomes (green tea, broccoli, lemon) can directly shape intestinal flora and maintain immune homeostasis through oral administration, thereby preventing and treating various intestinal diseases (Teng et al., 2018; Lei et al., 2020; Zu et al., 2021). Therefore, inspired researchers began to try to apply plant extracellular vesicles to tumor cells. It is gratifying that these vesicles also showed their good anticancer activities, Including inhibiting the proliferation and invasion of malignant tumors, promoting the apoptosis of cancer cells, inhibiting the cell cycle, and reducing the drug resistance of cancer cells (Friedmann, 2016; Cao et al., 2019; Potesta et al., 2020; Stanly et al., 2020; Yang et al., 2021; Zhang et al., 2021).

Melanoma, a deadly skin disease, is a major public health problem in many white countries (Jackett and Scolyer, 2019). UV radiation is often considered a trigger, and increased UV exposure increases the incidence of melanoma (Keim et al., 2021). Ginseng is a traditional Chinese medicine in Southeast Asian countries, in addition to ginsenosides exhibiting tumor-suppressive effects in the treatment of melanoma, Ginseng extracellular vesicles isolated from ginseng cell culture supernatants improve cellular melanosis induced by UV-B radiation by downregulating melanin-producing proteins (Zhou and Yang, 2015; Meng et al., 2019; Cho et al., 2021). Ginseng extracellular vesicles, dependent on TLR4 and MyD88 signaling, promote the transformation of tumor-associated macrophages from the M2 phenotype to the M1 phenotype and inhibit the growth of melanoma (Cao et al., 2019). Extracellular vesicles extracted from the leaves and stems of Dendrobium inhibited the expression of the melanin production-related gene MITF and the tyrosine-related proteins TYR, TRP-1 and TRP-2, inducing melanin reduction in melanoma cells (Lee et al., 2020). In addition to ginseng extracellular vesicles, grapefruit extracellular vesicles have been shown to reduce the expression levels of cyclins B1 and B2 and upregulate the expression of the cell cycle inhibitor P21, arrest the melanoma cell cycle at the G2/M point, and inhibit melanin Proliferation of tumor cells (Stanly et al., 2020). Cervical cancer caused by human papillomavirus has an extremely high fatality rate in less developed regions. Vesicles isolated from aqueous extracts of *Moringa oleifera* seeds were shown to induce decreased tumor cell growth and increased apoptosis in cultured hela cells *in vitro* by downregulating B-cell lymphoma 2 anti-apoptotic proteins (Abdul Rahman et al., 2020; Potesta et al., 2020). In the research of liver cancer treatment, the research of asparagus extracellular vesicles has made new achievements. The results of *in vivo* and *in vitro* experiments showed that asparagus extracellular vesicles inhibited the proliferation of cancer cells by reducing the expression levels of Ki67 and PCNA in liver cancer cells, and increase the protein levels of AIF, Bax and Bak to trigger the activation of caspase-9, leading to cleavage of key cellular proteins including the DNA repair enzyme PARP and ultimately cancer cell death (Zhang et al., 2021). The down-regulation of apoptosis-related protein caspase 3 and the up-regulation of cleaved caspase 3 in the experiments of lemon extracellular vesicles for cancer treatment indicated that lemon extracellular vesicles could induce apoptosis of gastric cancer cells. In addition, gastric cancer cells undergo S-phase cell cycle arrest, and cancer cell proliferation is also inhibited (Yang et al., 2020). In addition, citrus lemon exosomes have also been shown to inhibit different tumor cell lines by specifically reaching the tumor site and activating TRAIL-mediated apoptotic cell death: A549 (human lung cancer cell line), SW480 (human colorectal adenocarcinoma) cell line), LAMA84 (chronic myeloid leukemia cell line) cancer cell proliferation (Raimondo et al., 2015). In mouse colon tumor cells, the researchers found that maize extracellular vesicles produced TNF-α by inhibiting colon 26 cell proliferation (direct effect) and by activating macrophages and other immune cells to infiltrate the tumor (indirect effect) to inhibit tumor growth in mice. The synergistic or additive effects of the two actions resulted in a significant *in vivo* antitumor effect of maize extracellular vesicles (Sasaki et al., 2021). Garlic-derived extracellular vesicles have anticancer properties against A498 (human renal carcinoma cell line), with reduced cell proliferation and cell cycle arrest in S phase. The expression levels of pro-apoptotic genes such as p53, Bax, Cas3 and Cas9 in tumor cells treated with garlic extracellular vesicles were significantly increased, and the expression levels of Bcl-2 anti-apoptotic genes were significantly decreased. As a potent angiogenic factor, VEGF secretion was also significantly reduced, adversely affecting tumor angiogenesis in cancer cells (Ozkan et al., 2021). In conclusion, plant extracellular vesicles can inhibit the proliferation and invasion of cancer cells, promote cell apoptosis, and inhibit cell cycle through immune pathways (macrophages and B lymphocytes) and direct effects. The detailed pathways of plant extracellular vesicles are shown in Table 1.

## Plant extracellular vesicles as drug delivery vehicles for antitumor

To date, the application of mammalian extracellular vesicles in cancer therapy has covered various aspects such as targeted chemotherapy, gene therapy and vaccine development (Liang et al., 2021; Tarasov et al., 2021). However, plant-derived extracellular vesicles have a similar structural composition to animal extracellular vesicles (Baldrich et al., 2019; Pocsfalvi et al., 2019; Stanly et al., 2019), yet only a few studies have developed them as a drug delivery platform. Compared with animal extracellular vesicles, plant extracellular vesicles are superior to animal-derived extracellular vesicles by virtue of their natural origin and the absence of zoonotic or human pathogens (De Jong and Borm, 2008; Dad et al., 2021). Plant extracellular vesicles have been proved to be safe and reliable through three routes of administration: oral, transdermal, and blood transport (Kusuma et al., 2016; Munir et al., 2020; Kim et al., 2021). Recent studies have shown that plant extracellular vesicles play an important role in the pathogenesis of various diseases, including cancer. Ginger exosomes have miRNAs that inhibit the expression of Nsp12 in lung epithelial cells and prevent Nsp12+-mediated lung inflammation in the treatment of inflammation (Teng et al., 2021). Ginger exosomes can be used to deliver the anti-inflammatory drug methotrexate for colitis and as an immunotherapeutic agent to maintain intestinal macrophage homeostasis (Wang et al., 2014). It is also used in Alzheimer’s disease and diabetes treatment by preventing abnormal activation of the NLRP3 (9). In the field of cancer treatment, new breakthroughs have also been made. It has been demonstrated that in combination with the chemotherapeutic drug 5-fluorouracil (5-FU), bitter melon exosomes enhance cytotoxicity and reduce 5-FU resistance during oral squamous cell carcinoma cell (OSCC) therapy by downregulating the expression of inflammasome NLRP3. Bitter melon extracellular vesicles induce apoptosis in oral squamous cell carcinoma cells by triggering ROS-mediated mitochondrial damage, a process possibly mediated by MAP30 protein (Yang et al., 2021). To Inhibit brain tumor progression, miR17 was encapsulated in grapefruit exosomes coated with folic acid and polyethylenimine, miR17 was rapidly delivered to the brain *via* the intranasal route and was selectively taken up by GL-26 cancer cells. Killed cancer cells by inhibiting MHC1 expression in GL-26 cancer cells and triggered the activation of NK cells (Friedmann, 2016). In addition to delivering miRNA therapeutics, some research groups have combined grapefruit extracellular vesicles with aptamer HA1 to load the chemotherapeutic drug azithromycin to target and kill HER2+ breast cancer cells (Tang et al., 2020). Coating grapefruit exosomes with membranes of activated leukocytes increases LFA-1 or CXCR1 and CXCR2 expression and improves the recruitment of grapefruit exosomes to cancer cells. Doxorubicin encapsulated in grapefruit exosomes was successfully delivered to the CT26 colon cancer and exerted tumoricidal effect (Wang et al., 2015). In addition, new research shows that in the treatment of ovarian cancer, functional heparin-cRGD (HR) was modified on the surface of lemon exosomes, and then the modified lemon exosomes were loaded with the drug doxorubicin through endocytosis into cancer cells. The expression of P-glycoprotein (P-gp) is inhibited, the production of ATP is reduced, the energy generated by ATP hydrolysis is reduced, and the excretion of drugs from cancer cells is reduced, thereby achieving the purpose of overcoming cancer drug resistance (Xiao et al., 2022). In summary, in practical applications, in addition to directly using the original plant extracellular vesicles as a drug delivery platform, in order to enhance its targeting, specific tumor-specific ability, and enhance tumor-related anti-tumor properties, researchers have also tried different approaches to redesign plant extracellular vesicles. The modification methods of each plant extracellular vesicle in this review are shown in Table 2.

## Discussion

Cancer is increasingly important as the leading cause of premature human death globally and, based on current trends, may even surpass cardiovascular disease as the leading cause of premature death in most countries, especially developing countries (Jiang et al., 2021). Accurate diagnosis and effective treatment of cancer is undoubtedly a huge problem in clinical treatment. In addition to the commonly used treatments such as gene therapy, immunotherapy, and chemotherapy (Vatner et al., 2014; Dayakar et al., 2022), researchers have attempted to develop extracellular vesicles as biomarker molecules and delivery platforms for cancer diagnosis and treatment (Pullan et al., 2019; Zhou et al., 2020). More and more studies have demonstrated that extracellular vesicles participate in and play an important role in multiple processes of cancer development. In addition to being an integral part of the plant’s own biological machinery and acting as a defense frontline during plant infection and growth and development, plant extracellular vesicles have also been developed as therapeutic agents to play a role in human health and disease (Rutter and Innes, 2018; Dad et al., 2021; Xu et al., 2022). The active substances such as proteins and nucleic acids contained in plant extracellular vesicles can maintain their physiological activities in recipient cells and affect the development of diseases (Baldrich et al., 2019; Chen et al., 2022). Plant extracellular vesicles can resist the activity of digestive enzymes in the gastrointestinal tract without being broken down, can be absorbed in the intestine, and can also function through the blood route (Munir et al., 2020). In addition to plant extracellular vesicles themselves acting as therapeutic particles, plant extracellular vesicles are also often designed as drug carriers to deliver drugs. Compared with delivery vehicles such as liposomes, silver nanoparticles and animal extracellular vesicles, plant extracellular vesicles are regarded as a natural source, biocompatibility, non-immunogenicity and non-toxicity to normal cells, an ideal nanosphere to hold and transport cargo (De Jong and Borm, 2008). It is also relatively easy to obtain plant extracellular vesicles, which can be obtained directly from plant stems and leaves, plant seeds and fruit juices (Rutter and Innes, 2017; Potesta et al., 2020; Bruno et al., 2021; Karimi et al., 2022). Although the application of plant extracellular vesicles seems to be easier and faster than that of animal extracellular vesicles, many efforts are still required. In addition, extensive immunogenicity studies are required to assess *in vivo* safety and efficacy before large-scale production use. In the face of tumor cells that may metastasize at any time, how to effectively load drugs, target delivery and release of drugs, deeply penetrate tumors and increase drug accumulation in tumor cells still need further research by scholars. Finally, the lack of specific markers still hinders the characterization of plant extracellular vesicles, although the omics analyses associated with them are increasing. Whether the source of plant extracellular vesicles is contaminated or not deserves further investigation.

## Conclusion

Plant extracellular vesicles play an important role in the treatment of diseases including cancer. Plant extracellular vesicles contain biologically active particles such as miRNA, proteins, and nucleic acids, which can interfere with the development of tumor cells. In addition, plant extracellular vesicles are also an ideal drug delivery vehicle, which can be modified for targeted delivery of anticancer drugs.
